# *trans*-2,5-Di­methyl­piperazine-1,4-diium bis(perchlorate) dihydrate: crystal structure and Hirshfeld surface analysis

**DOI:** 10.1107/S205698901600520X

**Published:** 2016-03-31

**Authors:** Cherifa Ben Mleh, Thierry Roisnel, Houda Marouani

**Affiliations:** a Laboratoire de Chimie des Matériaux, Faculté des Sciences de Bizerte, 7021 Zarzouna Bizerte, Université de Carthage, Tunisia; b Centre de Diffractométrie X, UMR 6226 CNRS, Unité Sciences Chimiques de Rennes, Université de Rennes I, 263 Avenue du, Général Leclerc, 35042 Rennes, France

**Keywords:** crystal structure, piperazine derivative, mol­ecular salt, hydrogen bonding, Hirshfeld surface analysis

## Abstract

The asymmetric unit of the title hydrated mol­ecular salt, C_6_H_16_N_2_^2+^·2ClO_4_^−^·2H_2_O, contains a half dication (completed by inversion symmetry), a perchlorate anion and a water mol­ecule. The extended structure consists of infinite chains of formula [(ClO_4_)H_2_O]*_n_^n^* ^−^ ions extending along the *b* axis linked by O_w_—H⋯O (w = water) hydrogen bonds. These chains are cross-linked by the dications *via* N—H⋯O_w_ and weak C—H⋯O hydrogen bonds, thus forming a three-dimensional supra­molecular network. Three-dimensional Hirshfeld surface analysis and two-dimensional fingerprint maps reveal that the structure is dominated by H⋯O/O⋯H and H⋯H contacts.

## Abstract 2

The extended structure consists of infinite [010] chains linked by O_w_—H⋯O (w = water) hydrogen bonds. These chains are cross-linked by the dications *via* N—H⋯O_w_ and weak C—H⋯O hydrogen bonds, thus forming a three-dimensional supra­molecular network. Three-dimensional Hirshfeld surface analysis and two-dimensional fingerprint maps reveal that the structure is dominated by H⋯O/O⋯H and H⋯H contacts.

## Chemical context   

Piperazine (C_4_H_10_N_2_) and its derivatives are a family of strongly basic amines able to form dications, in which all of the N—H bonds are generally active in hydrogen-bond formation. They are used in pharmacology and found in biologically active compounds across a number of different therapeutic areas, displaying anti­bacterial, anti­fungal, anti­malarial, anti­psychotic, anti­depressant and anti­tumor activity (Brockunier *et al.*, 2004 ▸; Bogatcheva *et al.*, 2006 ▸).
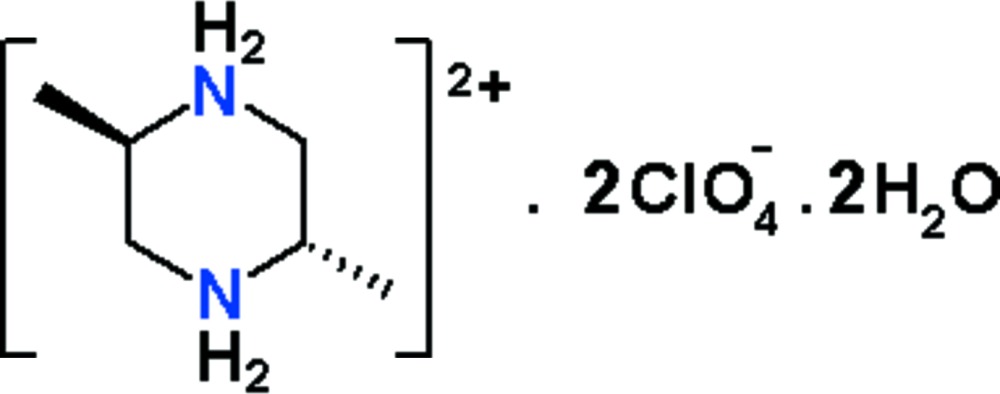


In this work, as part of our studies in this area, we report the preparation and structural investigation of a new hydrated perchlorate salt, C_6_H_16_N_2_^2+^·2ClO_4_^−^·2H_2_O (I).

## Structural commentary   

**Figure 1:**
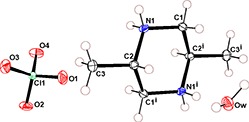
An *ORTEP* view of (I) with displacement ellipsoids drawn at the 30% probability level. Symmetry code: (i) −*x* + 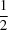, −*y* + 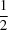, −*z*.

The asymmetric unit of (I) is composed of a half of a *trans*-2,5-dimethylpipeazine-1,4-dium dication, one perchlorate anion and one water mol­ecule (Fig. 1 ▸). The complete dication is generated by crystallographic inversion symmetry, leading to a typical chair conformation, with the methyl groups occupying equatorial positions [puckering parameters: *Q* = 0.7341 Å, θ = 90 and φ = −16 °], which is similar the conformation of the same species in its nitrate salt (Gatfaoui *et al.*, 2014 ▸). Otherwise, the bond lengths and angle in the dication are normal (Rother *et al.*, 1997 ▸; Gatfaoui *et al.*, 2014 ▸; Essid *et al.*, 2015 ▸).

The perchlorate anion displays its expected tetra­hedral geometry around the chlorine atom. Inter­atomic bond lengths and angles of the perchlorate anion lie respectively within the ranges [1.4327 (10)–1.4452 (11) Å] and [109.01 (7)- 110.28 (7) °]. Similar geometrical features have also been noticed in other crystal structures (Toumi Akriche *et al.*, 2010 ▸; Berrah *et al.*, 2012 ▸).

## Supra­molecular features   

**Figure 2:**
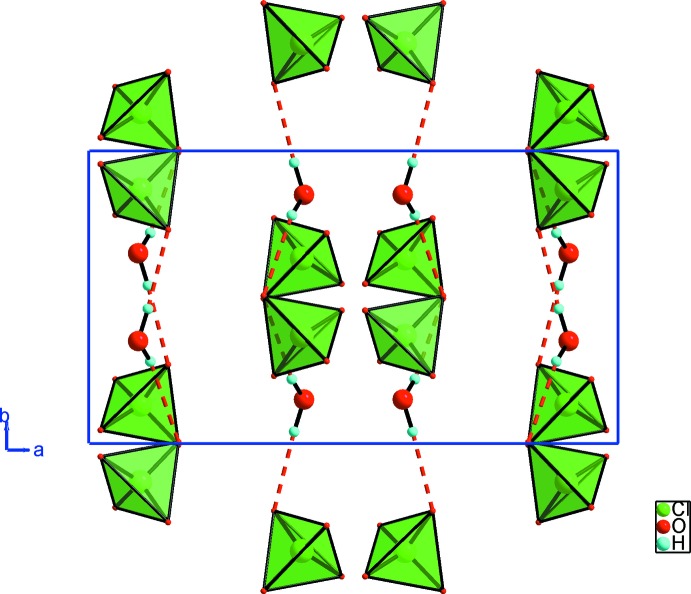
Hydrogen-bonded supra­molecular chains involving anions and water mol­ecules of compound (I), represented through the *ab* plane.

**Figure 3:**
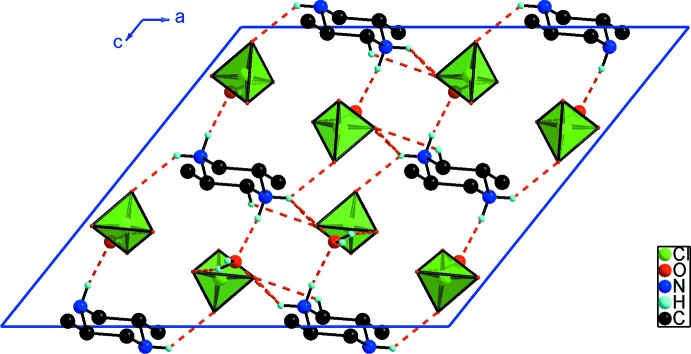
Projection of (I) along the *b* axis. The H-atoms not involved in hydrogen bonding are omitted.

In the extended structure, the anions are connected to the water mol­ecules through O_w_—H⋯O hydrogen bonds (Table 1 ▸), generating a corrugated 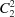
(5) chain running along the [010] direction (Fig. 2 ▸). These chains are linked *via* the *trans*-2,5-dimethlpiperazine-1,4-diium cations through N—H⋯O, N—H⋯O_w_ and weak C—H⋯O hydrogen bonds, forming a three-dimensional supra­molecular network (Fig. 3 ▸). These data show that each organic cation is connected to six inorganic chains.

## Hirshfeld surface analysis   

**Figure 4:**
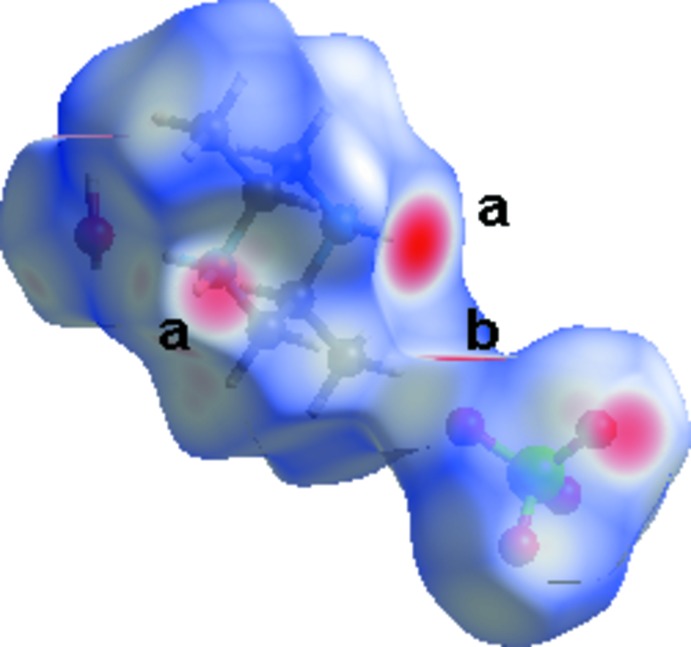
Hirshfeld surface around the constituents of (I) coloured according to *d*_norm_. The surfaces are shown as transparent to allow visualization of the orientation and conformation of the functional groups.

The three-dimensional Hirshfeld surfaces and two-dimensional fingerprint plots of (I) were prepared using *CrystalExplorer* (Wolff *et al.*, 2012 ▸) and are shown in Figs. 4 ▸ and 5 ▸, respectively. The inter­action between N—H and oxygen atoms can be seen in the Hirshfeld surface as the bright-red area in Fig. 4 ▸ (labeled *a*). The light-red spots are due to O_w_—H⋯O inter­actions (labeled *b*). For the salt, O⋯H/H⋯O contacts, which are attributed to N—H⋯O_w_ and O_w_—H⋯O hydrogen-bonding inter­actions, appear as two sharp symmetric spikes in the two-dimensional fingerprint maps. They have the most significant contribution to the total Hirshfeld surfaces. The H⋯H contacts appear in the middle of the scattered points in the two-dimensional fingerprint maps. For further information on Hirshfeld surfaces, see: Spackman & McKinnon (2002 ▸) and Spackman & Jayatilaka (2009 ▸).

## Synthesis and crystallization   

The title compound was prepared from an alcoholic solution containing *trans*-2,5-di­methyl­piparazine (0.1 g, 1 mmol, purity 99%, Aldrich) dissolved in ethanol (20 ml) and perchloric acid HClO_4_ (0.2 g, 2 mmol, purity 96%, Aldrich) with a molar ratio of 1:2. This mixture was stirred for 1 h. After a week of evaporation at room temperature, colorless single crystals of suitable dimensions for crystallographic study were formed, and were isolated by filtration and washed with a small amount of distilled water. The crystals can be stable for months under normal conditions of temperature and humidity.

## Refinement   

Crystal data, data collection and structure refinement details are summarized in Table 2 ▸. All H atoms were located in a difference map but were placed geometrically and refined using a riding model, with C—H = 0.96 Å (meth­yl), or 0.98 Å (methine), N—H = 0.90 Å (NH_2_) with *U*_iso_(H) = 1.2*U*_eq_(C or N). The H atoms of the water mol­ecule were refined with a distance restraint of O—H = 0.85 (1) Å using DFIX and DANG commands (Sheldrick, 2015 ▸) with *U*_iso_(H) = 1.5*U*_eq_(O).

## Supplementary Material

Crystal structure: contains datablock(s) I. DOI: 10.1107/S205698901600520X/hb7574sup1.cif

Structure factors: contains datablock(s) I. DOI: 10.1107/S205698901600520X/hb7574Isup2.hkl

CCDC reference: 1470800

Additional supporting information:  crystallographic information; 3D view; checkCIF report

## Figures and Tables

**Figure 5:**
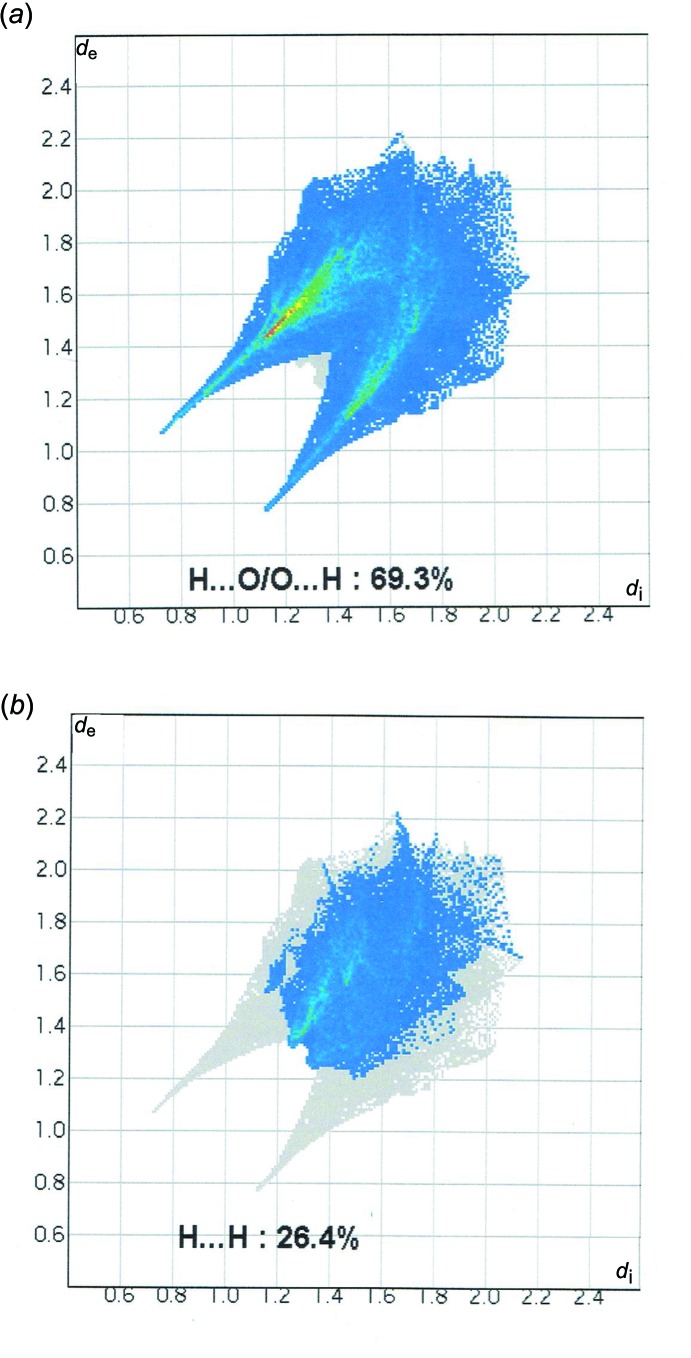
Fingerprint plots of the major contacts: (*a*) H⋯O and (*b*) H⋯H.

**Table 1:** Hydrogen-bond geometry (Å, °)

*D*—H⋯*A*	*D*—H	H⋯*A*	*D*⋯*A*	*D*—H⋯*A*
O*W*—H1*W*⋯O1^i^	0.85 (1)	2.03 (1)	2.8637 (16)	167 (2)
O*W*—H2*W*⋯O2^ii^	0.85 (1)	2.23 (1)	2.9932 (16)	150 (2)
N1—H1*N*⋯O4^iii^	0.90	2.18	2.9067 (15)	137
N1—H1*N*⋯O3^iv^	0.90	2.42	3.0293 (15)	125
N1—H1*N*⋯O*W* ^v^	0.90	2.55	3.1994 (16)	130
N1—H2*N*⋯O*W* ^i^	0.90	1.91	2.8019 (15)	172
C1—H1*B*⋯O3^iv^	0.97	2.56	3.1007 (17)	116

Symmetry codes: (i) 
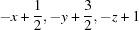
; (ii) 
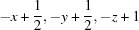
; (iii) 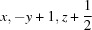; (iv) 
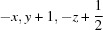
; (v) 
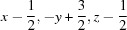
.

**Table 2:** Experimental details

Crystal data	
Chemical formula	C_6_H_16_N_2_^2+^·2ClO_4_^−^·2H_2_O
*M*_r_	351.14
Crystal system, space group	Monoclinic, *C*2/*c*
Temperature (K)	150
*a*, *b*, *c* (Å)	16.8603 (8), 7.2655 (3), 14.4534 (6)
β (°)	128.751 (1)
*V* (Å^3^)	1380.78 (10)
*Z*	4
Radiation type	Mo *K*α
μ (mm^−1^)	0.52
Crystal size (mm)	0.44 × 0.29 × 0.25

Data collection	
Diffractometer	Bruker D8 VENTURE
Absorption correction	Multi-scan (*SADABS*; Bruker, 2014 ▸)
*T*_min_, *T*_max_	0.775, 0.878
No. of measured, independent and observed [*I* > 2σ(*I*)] reflections	7760, 1557, 1457
*R*_int_	0.023
(sin θ/λ)_max_ (Å^−1^)	0.649

Refinement	
*R*[*F* ^2^ > 2σ(*F* ^2^)], *wR*(*F* ^2^), *S*	0.028, 0.074, 1.13
No. of reflections	1557
No. of parameters	100
No. of restraints	3
H-atom treatment	H atoms treated by a mixture of independent and constrained refinement
Δρ_max_, Δρ_min_ (e Å^−3^)	0.34, −0.41

Computer programs: *APEX2* and *SAINT* (Bruker, 2014 ▸), *SIR97* (Altomare *et al.*, 1999 ▸), *SHELXL2014/7* (Sheldrick, 2015 ▸), *ORTEP-3 for Windows* and *WinGX* publication routines (Farrugia, 2012 ▸).

## supplementary crystallographic information

### Crystal data

**Table:**

C_6_H_16_N_2_^2+^·2ClO_4_^−^·2H_2_O	*F*(000) = 736
*M**_r_* = 351.14	*D*_x_ = 1.689 Mg m^−^^3^
Monoclinic, *C*2/*c*	Mo *K*α radiation, λ = 0.71073 Å
*a* = 16.8603 (8) Å	Cell parameters from 7552 reflections
*b* = 7.2655 (3) Å	θ = 3.1–27.5°
*c* = 14.4534 (6) Å	µ = 0.52 mm^−^^1^
β = 128.751 (1)°	*T* = 150 K
*V* = 1380.78 (10) Å^3^	Prism, colourless
*Z* = 4	0.44 × 0.29 × 0.25 mm

### Data collection

**Table:**

D8 VENTURE Bruker AXS diffractometer	1557 independent reflections
Radiation source: Incoatec microfocus sealed tube	1457 reflections with *I* > 2σ(*I*)
Multilayer monochromator	*R*_int_ = 0.023
rotation images scans	θ_max_ = 27.5°, θ_min_ = 3.1°
Absorption correction: multi-scan (*SADABS*; Bruker, 2014)	*h* = −21→21
*T*_min_ = 0.775, *T*_max_ = 0.878	*k* = −9→9
7760 measured reflections	*l* = −18→15

### Refinement

**Table:**

Refinement on *F*^2^	Primary atom site location: structure-invariant direct methods
Least-squares matrix: full	Secondary atom site location: difference Fourier map
*R*[*F*^2^ > 2σ(*F*^2^)] = 0.028	Hydrogen site location: inferred from neighbouring sites
*wR*(*F*^2^) = 0.074	H atoms treated by a mixture of independent and constrained refinement
*S* = 1.13	*w* = 1/[σ^2^(*F*_o_^2^) + (0.0308*P*)^2^ + 1.9533*P*] where *P* = (*F*_o_^2^ + 2*F*_c_^2^)/3
1557 reflections	(Δ/σ)_max_ = 0.001
100 parameters	Δρ_max_ = 0.34 e Å^−^^3^
3 restraints	Δρ_min_ = −0.41 e Å^−^^3^

### Special details

**Table:**

Geometry. All e.s.d.'s (except the e.s.d. in the dihedral angle between two l.s. planes) are estimated using the full covariance matrix. The cell e.s.d.'s are taken into account individually in the estimation of e.s.d.'s in distances, angles and torsion angles; correlations between e.s.d.'s in cell parameters are only used when they are defined by crystal symmetry. An approximate (isotropic) treatment of cell e.s.d.'s is used for estimating e.s.d.'s involving l.s. planes.
Refinement. Refinement of *F*^2^ against ALL reflections. The weighted *R*-factor *wR* and goodness of fit *S* are based on *F*^2^, conventional *R*-factors *R* are based on *F*, with *F* set to zero for negative *F*^2^. The threshold expression of *F*^2^ > σ(*F*^2^) is used only for calculating *R*-factors(gt) *etc*. and is not relevant to the choice of reflections for refinement. *R*-factors based on *F*^2^ are statistically about twice as large as those based on *F*, and *R*- factors based on ALL data will be even larger.

### Fractional atomic coordinates and isotropic or equivalent isotropic displacement parameters (Å^2^)

**Table:**

	*x*	*y*	*z*	*U*_iso_*/*U*_eq_	
Cl1	0.09748 (2)	0.13197 (4)	0.16201 (3)	0.01195 (12)	
O1	0.14991 (10)	0.26958 (16)	0.25415 (10)	0.0307 (3)	
O2	0.16959 (8)	−0.00473 (16)	0.18348 (11)	0.0254 (3)	
O3	0.02214 (9)	0.04530 (16)	0.16384 (10)	0.0239 (3)	
O4	0.05035 (9)	0.21898 (16)	0.04934 (9)	0.0236 (3)	
OW	0.40676 (9)	0.84994 (14)	0.78311 (9)	0.0203 (2)	
H1W	0.3881 (18)	0.9600 (15)	0.780 (2)	0.046 (7)*	
H2W	0.3837 (15)	0.780 (2)	0.8086 (18)	0.034 (6)*	
N1	0.14181 (8)	0.75580 (15)	0.43347 (10)	0.0111 (2)	
H2N	0.1201	0.7208	0.3612	0.013*	
H1N	0.0867	0.7774	0.4286	0.013*	
C1	0.20218 (10)	0.92935 (18)	0.46870 (12)	0.0119 (3)	
H1A	0.2220	0.9720	0.5442	0.014*	
H1B	0.1605	1.0239	0.4099	0.014*	
C2	0.20319 (10)	0.60230 (18)	0.52072 (11)	0.0114 (3)	
H2	0.2243	0.6396	0.5984	0.014*	
C3	0.13915 (11)	0.42938 (19)	0.48147 (13)	0.0186 (3)	
H3A	0.0801	0.4542	0.4754	0.028*	
H3B	0.1785	0.3334	0.5385	0.028*	
H3C	0.1183	0.3911	0.4056	0.028*	

### Atomic displacement parameters (Å^2^)

**Table:**

	*U*^11^	*U*^22^	*U*^33^	*U*^12^	*U*^13^	*U*^23^
Cl1	0.01181 (18)	0.01242 (18)	0.01379 (18)	−0.00022 (10)	0.00907 (14)	0.00166 (10)
O1	0.0321 (6)	0.0200 (6)	0.0225 (6)	−0.0075 (5)	0.0085 (5)	−0.0069 (5)
O2	0.0215 (6)	0.0256 (6)	0.0347 (6)	0.0119 (5)	0.0203 (5)	0.0101 (5)
O3	0.0246 (6)	0.0246 (6)	0.0358 (6)	−0.0064 (4)	0.0253 (5)	−0.0012 (5)
O4	0.0269 (6)	0.0308 (6)	0.0190 (5)	0.0105 (5)	0.0171 (5)	0.0119 (4)
OW	0.0272 (6)	0.0152 (5)	0.0174 (5)	−0.0013 (4)	0.0135 (5)	0.0000 (4)
N1	0.0077 (5)	0.0131 (5)	0.0124 (5)	0.0006 (4)	0.0062 (4)	0.0008 (4)
C1	0.0120 (6)	0.0101 (6)	0.0142 (6)	0.0011 (5)	0.0084 (5)	0.0006 (5)
C2	0.0110 (6)	0.0113 (6)	0.0122 (6)	0.0008 (5)	0.0074 (5)	0.0020 (5)
C3	0.0163 (6)	0.0139 (6)	0.0230 (7)	−0.0034 (5)	0.0111 (6)	0.0008 (5)

### Geometric parameters (Å, º)

**Table:**

Cl1—O3	1.4327 (10)	C1—C2^i^	1.5218 (17)
Cl1—O4	1.4363 (10)	C1—H1A	0.9700
Cl1—O1	1.4425 (11)	C1—H1B	0.9700
Cl1—O2	1.4452 (11)	C2—C3	1.5163 (18)
OW—H1W	0.850 (9)	C2—C1^i^	1.5218 (17)
OW—H2W	0.850 (9)	C2—H2	0.9800
N1—C1	1.4955 (16)	C3—H3A	0.9600
N1—C2	1.5071 (16)	C3—H3B	0.9600
N1—H2N	0.9000	C3—H3C	0.9600
N1—H1N	0.9000		
			
O3—Cl1—O4	110.28 (7)	N1—C1—H1B	109.5
O3—Cl1—O1	109.01 (7)	C2^i^—C1—H1B	109.5
O4—Cl1—O1	109.03 (7)	H1A—C1—H1B	108.1
O3—Cl1—O2	109.29 (7)	N1—C2—C3	110.17 (10)
O4—Cl1—O2	109.87 (7)	N1—C2—C1^i^	108.88 (10)
O1—Cl1—O2	109.34 (7)	C3—C2—C1^i^	111.63 (11)
H1W—OW—H2W	109.1 (17)	N1—C2—H2	108.7
C1—N1—C2	111.99 (10)	C3—C2—H2	108.7
C1—N1—H2N	109.2	C1^i^—C2—H2	108.7
C2—N1—H2N	109.2	C2—C3—H3A	109.5
C1—N1—H1N	109.2	C2—C3—H3B	109.5
C2—N1—H1N	109.2	H3A—C3—H3B	109.5
H2N—N1—H1N	107.9	C2—C3—H3C	109.5
N1—C1—C2^i^	110.74 (10)	H3A—C3—H3C	109.5
N1—C1—H1A	109.5	H3B—C3—H3C	109.5
C2^i^—C1—H1A	109.5		

Symmetry code: (i) −*x*+1/2, −*y*+3/2, −*z*+1.

### Hydrogen-bond geometry (Å, º)

**Table:**

*D*—H···*A*	*D*—H	H···*A*	*D*···*A*	*D*—H···*A*
O*W*—H1*W*···O1^i^	0.85 (1)	2.03 (1)	2.8637 (16)	167 (2)
O*W*—H2*W*···O2^ii^	0.85 (1)	2.23 (1)	2.9932 (16)	150 (2)
N1—H1*N*···O4^iii^	0.90	2.18	2.9067 (15)	137
N1—H1*N*···O3^iv^	0.90	2.42	3.0293 (15)	125
N1—H1*N*···O*W*^v^	0.90	2.55	3.1994 (16)	130
N1—H2*N*···O*W*^i^	0.90	1.91	2.8019 (15)	172
C1—H1*B*···O3^iv^	0.97	2.56	3.1007 (17)	116

Symmetry codes: (i) −*x*+1/2, −*y*+3/2, −*z*+1; (ii) −*x*+1/2, −*y*+1/2, −*z*+1; (iii) *x*, −*y*+1, *z*+1/2; (iv) −*x*, *y*+1, −*z*+1/2; (v) *x*−1/2, −*y*+3/2, *z*−1/2.
